# Comparative Evaluation of Effect of Micro-Osteoperforation and Mechanical Vibration on Rate of Orthodontic Tooth Movement in Young Adults With Bimaxillary Protrusion

**DOI:** 10.7759/cureus.36636

**Published:** 2023-03-24

**Authors:** Supriya S Wagh, Amit Nehete, Nitin Gulve, Shivpriya Aher, Digvijay Patil, Mukul Tambe

**Affiliations:** 1 Orthodontics and Dentofacial Orthopaedics, Mahatma Gandhi Vidyamandir's Karmaveer Bhausaheb Hiray Dental College & Hospital, Nashik, IND

**Keywords:** bimaxillary protrusion, tooth movement, canine retraction, mechanical vibration, micro-osteoperforation

## Abstract

Aim: To evaluate and compare the rate of orthodontic tooth movement and root resorption by micro-osteoperforation (MOP) and mechanical vibration in young adults with bimaxillary protrusion.

Method: Twenty patients having class I bimaxillary protrusion who required all first premolar extraction were allocated into two groups MOP (Group A) and mechanical vibration (Group B), with a 1:1 allocation ratio. After leveling alignment MOP was performed on either side of the arch, and vibration was applied on the contralateral side 20 mins per day. Canines were retracted with nickel-titanium coil springs, and Alginate impressions were taken every four weeks till 4 months.

Result: The mean rate of retraction of canines of Group A was more than Group B. There was a statistically significant difference between Group A and Group B. (p=0.0120)

Conclusion: The mean rate of retraction of canines treated by MOP was 1.15 mm per 4 weeks, and by mechanical vibration, 0.8mm per 4 weeks.

## Introduction

The orthodontic treatment enhances patients' esthetics, function, and self-esteem by correcting malocclusion. With an increasing number of adults seeking treatment for the correction of malocclusion and poor facial esthetics, a significant challenge in orthodontics is decreasing treatment time without compromising the treatment outcome [1]. Prolonged fixed orthodontic treatment is associated with adverse sequelae such as increased gingivitis, periodontitis, caries incidences, and external apical root resorption [2]. As a result, one of the critical difficulties in orthodontic research is finding a way to shorten the length of treatment without compromising the outcome [2].

Various methods are available in orthodontics for accelerating tooth movements, including invasive, minimally invasive, and non-invasive [3]. Among non-invasive methods, resonance vibration has gained popularity due to its noninvasiveness, portability, and ease of use [4]. A study by Leethanakul et al. investigated that applying vibratory stimuli with an electric toothbrush (125HZ) increased the secretion of interleukin 1β during canine retraction, indicative of accelerated tooth movement [5]. Harold Frost recognized in 1983 that molecular dynamics of osteogenesis in stressed bone is based on the Regional Acceleratory Phenomenon (RAP) rather than bony block movement. RAP causes a reduction in local bone density. He described RAP as a local response to a noxious stimulus. In minimal invasive methods, micro-osteoperforation (MOP) has shown promising results. It induces regional acceleratory phenomenon, producing local osteopenia, thereby accelerating tooth movement [6,7].

Though individual studies are available, there is no evidence in the literature where vibrations and MOPs are compared in the same study subjects. Therefore, this study aimed to evaluate and compare the rate of orthodontic tooth movement and root resorption by MOP and mechanical vibration in young adults with bimaxillary protrusion.

## Materials and methods

Study design

The study was a split-mouth, with a 1:1 allocation ratio designed to compare the orthodontic tooth movement rate with vibrations and MOP. The Research Ethics Committee of MGV's KBH dental college and hospital Nashik approved the study procedure. After obtaining ethical clearance from the institutional ethical committee, twenty patients indicated for all first premolar extractions were selected from the patients approaching the Department of Orthodontics and Dentofacial orthopedics with the help of the following criteria.

Inclusion and exclusion criteria

Adults aged 18 to 24 years, class I malocclusion with a bimaxillary protrusion, full permanent dentition excluding third molars, good dental hygiene, and periodontal health were used as the inclusion criteria for selecting the participants. Patients with a medically compromised condition, a history of long-term use of orthodontic tooth movement (OTM) -affecting medicines, smoking, and any radiographic indications of bone loss were excluded.

For each participant, by tossing a coin, the MOP intervention was randomly assigned to either the right or left side, while the opposite side served as a vibration group. Maxillary and mandibular quadrants assigned for MOP were included in Group A, and contralateral quadrants assigned for mechanical vibration were included in Group B.

Sample size calculation

The sample size calculation was based on a previous study investigating monthly canine retraction rates. Level of significance = 5%, Power = 80%, Type of test = two-sided. The formula for calculating sample size is the Sample size for the study (outcome variable on ratio scale) and testing null hypothesis: M1=M2 (means of two intervention groups). The M1-Estimated mean rate of space closure per month with micro osteoperforation (Group 1) was 1.5 mm, and M2- The estimated mean rate of space closure per month with mechanical vibration (Group 2) was 1.00 mm. 1-α set confidence level was0.95, and 1-β Set level of test power was0.8. Assuming all the factors, our sample size comes to around 20 per group (n1). The total sample size is 20 subjects considering it is a spilled mouth study.

Intervention

After obtaining diagnostic records and written consent, patients were referred for extraction of all first premolars. It was ensured that the same operator did extractions. After an interval of 1-week post-extraction, all teeth were bonded with a 0.022 slot MBT prescription bracket system. A power hook was directly bonded to the center of the labial surface of the canine to apply force close to the center of resistance. Standard archwire sequence was followed and after accomplishing leveling and alignment with 0.019 × 0.025-inch stainless steel archwire, mini-screws (1.5× 8 mm) were placed between 2nd premolar and 1st molar bilaterally in both the arches for anchorage purpose. Then the study model was obtained at T0 time, i.e., just before initiating canine retraction.

**Figure 1:**
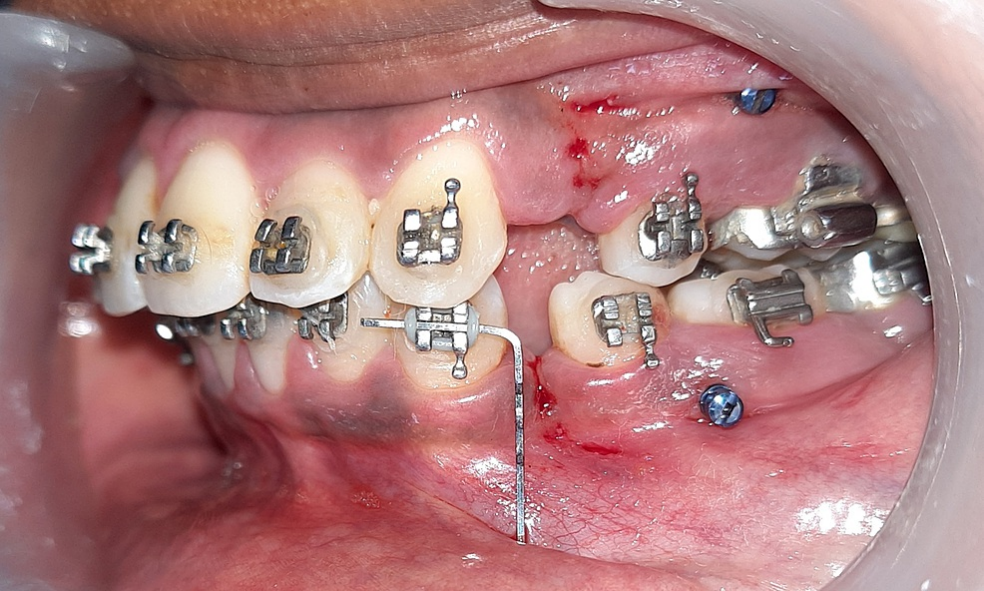
MOP done in the maxillary and mandibular arch. MOP: Micro-osteoperforation

Before initiating canine retraction, on the MOP side, an L-shaped wire guide (L- pin Guide) with its vertical segment equal to two-thirds of the canine root length was ligated to the canine bracket. The vernier caliper measured the distance between the canine bracket's distal tie wing and the premolar bracket's mesial tie wing. MOP was performed at the midpoint of this distance-vertical wire guide into three segments measuring 5mm, 2mm, and 2mm, respectively. Patients were asked to rinse their mouths with chlorhexidine for 1 minute. Then three MOPs were performed in the middle of the extraction space using L- a pin guide under local anesthesia (2% lidocaine with 1:1,00,000 epinephrine). MOPs were performed using a hand-held device loaded in the implant driver by placing a rubber stopper at a depth of 3 mm, resulting in perforations that were each 1.5 mm wide and 3 mm deep (figure 1). Temporary anchorage device (TAD) was screwed slowly into the alveolar bone, perpendicular to the bone surface then the TAD was unscrewed and removed.

**Figure 2:**
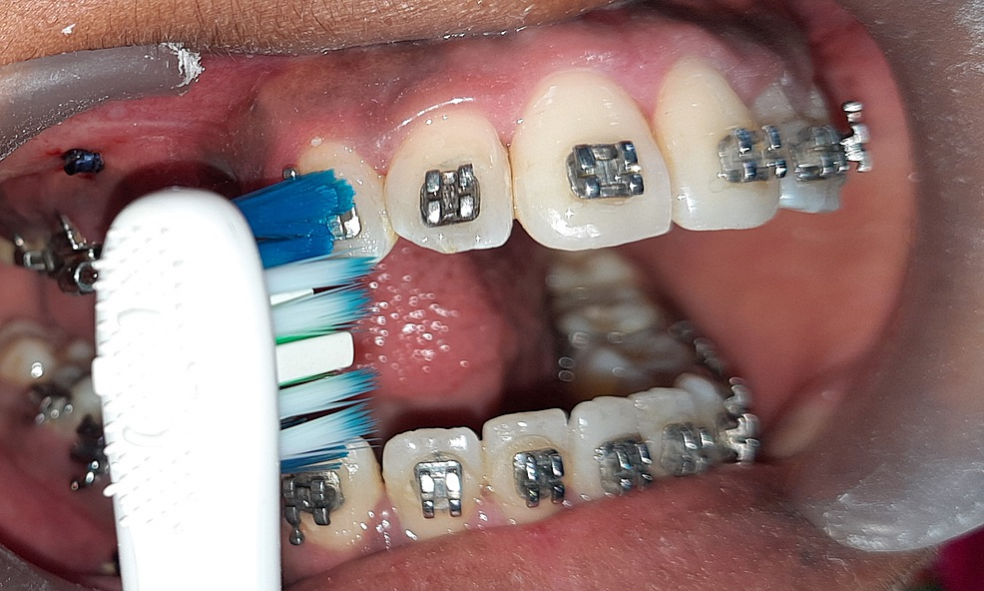
Application of vibration through a powered toothbrush.

Whereas on the other side, patients were instructed to hold an ultrasonic toothbrush with a frequency of 125 HZ on the labial surface of the canine for mechanical vibrations (Figure 2). Patients were advised to use it once a day for 20 mins. NiTi closed coil spring was stretched between TAD and the power hook on canines for initiating canine retraction with 100 gms force. Patients were checked at the 4-week interval, and every 4-week spring was activated to maintain force consistency.

Data collection

Follow-up visits were scheduled at 4 weeks of intervals. RAP continues approx four months after stimulation. That is why the study was terminated at the end of 16 weeks. For each patient, five study models were obtained starting from baseline time, i.e., T0 up to T4 (at the termination of the study). The following measurements were used in the study.

Rate of retraction of canine

**Figure 3:**
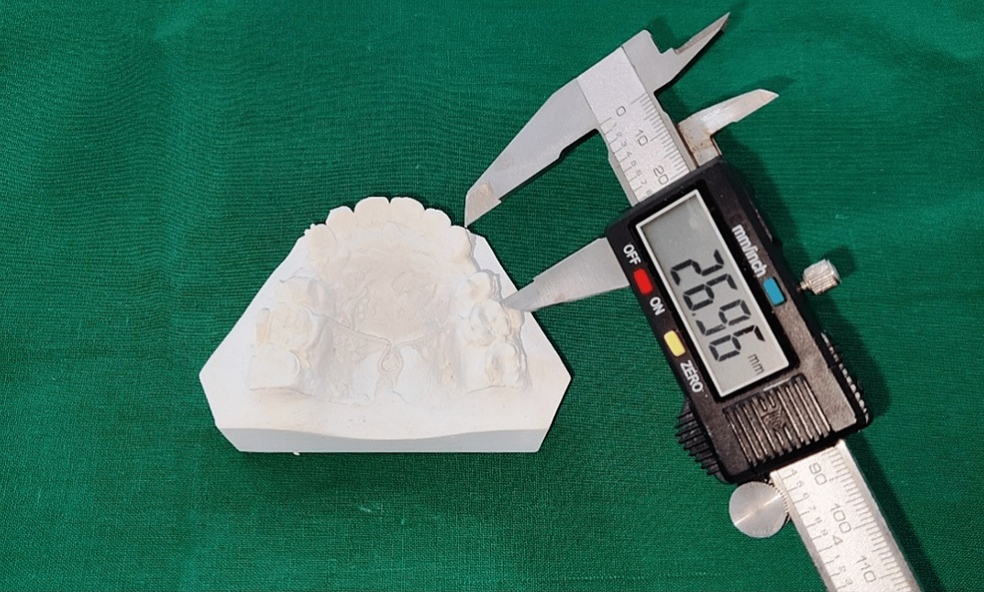
Measuring the distance from the canine cusp tip to the mesiobuccal cusp tip of first molar with the vernier caliper.

With the help of a digital vernier (least count 0.01mm), Caliper distance (D) was measured from the canine cusp tip to the tip of the mesiobuccal cusp of the first molar on all the study models (Figure 3). D0, D1, D2, D3, and D4 are the distances measured on study models obtained at times T0, T1, T2, T3, and T4, respectively. Then the rate of retraction of canine (R), i.e., distance moved by the canine in 4 weeks, at times T1, T2, T3, and T4, were obtained as follows-

R1= D0-D1

R2= (D0-D2) -(D0-D1)

R3=(D0-D3) -(D0-D2)

R4= (D0-D4) -(D0-D3)

Root resorption of canine

For evaluation of root resorption, OPG was obtained after the termination of the study, i.e., at T4. Root resorption was assessed on the maxillary and mandibular canines for both the groups using a four-grade ordinal scale proposed by Scott McNab as mentioned below [8]:

0 = no apical RR.

1 = slight blunting of the root apex.

2 = moderate resorption of root apex beyond blunting and up to one-third of the root length.

3 = severe resorption of root apex beyond one-third of root length.

Data obtained were subjected to statistical analysis.

Statistical analysis

Descriptive and inferential statistics were done using SPSS 0.17 version, and the significance level was set at < 0.05. With the help of descriptive statistics, mean values of the rate of canine retraction and frequency and percentage of root resorption of canine were obtained for both Group A and Group B. Whereas, with inferential statistics, mean values of the rate of retraction of canine and mean rank root resorption of canine for both the groups were compared. Inter-Group and intragroup comparisons were made using the unpaired t-test, Mann-Whitney U test, and One-way Anova F test followed by the Post hoc Tukey test. The following results were obtained from the study.

## Results

Rate of canine retraction

As shown in Table 1, it was observed that the mean rate of canine retraction of Group A at R1, R2, R3, and R4 was more than Group B. The comparison showed a statistically significant difference at R1, R2, and R3 between Group A and Group B. (p= 0.0007, 0.0026, and 0.0386, respectively). Also, no statistically significant difference was found at R4 between Group A and Group B (p=0.6129).

**Table 1:** Comparison of the rate of retraction of canines of Group A and Group B from T0 to T4.

Rate of canine retraction.	Micro-osteoperforation (Group A) Mean (S.D.)	Mechanical Vibration Group B) Mean (S.D.)	Unpaired t-test	P value, Significance
R1	1.40 (1.05)	0.8 (0.2)	t = 3.538	p = 0.0007**
R2	1.25 (0.39)	0.92 (0.52)	t = 3.112	p =0.0026*
R3	1.15 (0.26)	0.88 (0.78)	t = 2.104	p =0.0386*
R4	0.80 (0.34)	0.76 (0.39)	t = 0.5080	p =0.6129

As shown in (Table 2), the mean rate of retraction of canines of Group A was more than Group B. The comparison showed a statistically significant difference between Group A and Group B (p=0.0120).

**Table 2:** Comparison of the mean rate of retraction of canines of Group A and Group B.

Mean rate of retraction of canine of Group A	Mean rate of retraction of canine of Group B	Unpaired t-test	P value, significance
1.15 ± 0.52	0.84 ± 0.63	4.783	0.0120

Root resorption

90% of canines showed grade 0, i.e., absence of resorption in Group A, and 92.5% of canines showed grade 0 root resorption in Group B. Whereas grade 2 and grade 3 were not observed in both the groups (Table 3).

**Table 3:** Frequency and percentage of root resorption of canine using ordinal scale by McNab of Group A and Group B.

Root Resorption	Frequency Group A	Percentage (%) Group A	Frequency Group B	Percentage (%) Group B
0	36	90.0%	37	92.5%
1	4	10.0%	3	7.5%
2	0	0.0%	0	0.0%
3	0	0.0%	0	0.0%
Total	40	100%	40	100%

The mean rank of root resorption of canines in Group A was more than in Group B. The comparison showed no statistically significant difference between Group A and Group B. (p=0.694) (Table 4).

**Table 4:** Intergroup comparison of root resorption of canine of Group A and Group B

Group	N	Mean Rank	Sum of Rank	Mean Whitney U Statistic	Z	P value
Root Resorption in Group A at T4	40	41	1640	78.0	-0.393	0.694 Non Significant
Root Resorption in Group B at T4	40	40	1640			

## Discussion

The average duration of orthodontic treatment is 2 years. Longer duration of treatment not only increases the risk of gingivitis, root resorption, and enamel demineralization, but most importantly, it can affect patient compliance [9]. Accelerated orthodontics has become very popular in recent research work. Many techniques have been suggested to accelerate tooth movement, including invasive, minimally invasive, and non-invasive [3]. Among non-invasive methods, resonance vibration has gained popularity due to its noninvasiveness, portability, and ease of use [4]. Vibration in orthodontics has been applied to increase the rate of orthodontic tooth movement by accelerating the periodontal and bony tissue modeling and remodeling processes [10].

Leethanakul et al. investigated that applying vibratory stimuli with an electric toothbrush (125HZ) Increased interleukin-1β secretion during canine retraction indicating accelerated tooth movement [5]. Recently, the acceleratory method of MOP has been suggested. This procedure involves a flapless bone puncture, unlike a corticotomy. Teixeira et al., who first proposed this technique, postulated that small cortical bone perforations were sufficient to trigger RAP [11]. Though individual studies are available, there is no evidence in the literature where vibrations and MOPs are compared in the same study subjects. Therefore, the present study aimed to evaluate and compare the rate of orthodontic tooth movement and root resorption by MOP and mechanical vibration in young adults with bimaxillary protrusion. A total of 20 patients participated in the split-mouth randomized controlled trial. The participants were randomly assigned to two equal groups. The split-mouth study design was used. The main advantage of this design is the reduced biological variables and therefore requiring a smaller sample size.

Age was an essential factor in the rate of tooth movement, as Wilcko et al. demonstrated in their study. The osteoclast recruitment/activation rate or bone density has been linked to this factor. Only adults in the age group of 15 and 25 years were chosen, and the average age in both groups was identical to remove age's effect on tooth movement [12-13]. The timing of tooth extractions can also affect the rate of tooth movement by increasing the activity of inflammatory factors, which could obscure the assessment of the impact of MOPs. Six months were considered between the tooth extraction and MOPs to lessen this interference. The teeth were leveled and aligned during this time, and each archwire was sequenced to 0.019 × 0.025 inches of stainless steel.

In a human clinical trial, Alikhani et al. assessed the Propel device with a 1.5mm diameter and variable depth of 3-7mm to accelerate tooth movement. In recent work, Cheung et al. used the Propel device for MOP on the mesial and distal sides of premolars to the depth of 5 mm and measuring 1.5 mm in diameter. They evaluated their effects on root resorption [14]. Hence, miniscrew implants were used to create MOP with minimal trauma. Alikhani et al. tested the effect of 1,2,3 and 4 MOP on the rate of tooth movement, while 3 and 4 MOP could be used to achieve an accelerated tooth movement; thus, 3 MOPs were done, which was the number used in previous studies [1].

In this study, using an already available instrument for tooth cleaning, the powered toothbrush generates the oscillatory pattern, creating vibratory motions to the brush head that result in up-regulation mechanical signals for alveolar bone remodeling. On using Acceledent as a vibratory stimulus for accelerating tooth movement, it is necessary to visit the clinic frequently to apply vibrations. So we decided to give vibrations by the powered toothbrush. It was comfortable for the patient to give 20 mins vibration at home. Also, we asked the patients to apply gentle pressure from the powered toothbrush so it could not adversely affect hard tissue structures.

Though OPG is considered inferior in assessing root resorption, the radiation exposure by CBCT is higher than OPG. Due to ethical concerns, OPG was advised for root resorption to keep minimum radiation exposure.

As shown in table 1, the present study investigated that the mean rate of retraction of canine of group A at R1, R2, R3, and R4 is more than Group B. The comparison showed a statistically significant difference between Group A and B at R1, R2, and R3. This was because MOP induces inflammatory markers by RAP and accelerates the tooth movement, whereas the vibratory stimuli do not increase inflammatory markers. Also, no statistically significant difference was found at R4 because inflammatory markers decreased with time. Upon comparing the mean canine retraction rate in the MOP group, it was found to be almost identical (1.2mm/month), supporting the findings of Parihar et al. [15].

In the study conducted by Sivarajan et al., a slight difference was observed between the rate of canine retraction with MOPs and the control group. The rate of canine retraction was 4.16 (1.62 mm) with MOPs and 3.06 (1.64 mm) with the control group over a 16-week observation period [16]. Moreover, Alikhani et al. found that the rate of tooth movement was 2.3 times higher in the experimental group by using three MOPs on the buccal cortex of an extracted first premolar for canine retraction. In contrast, Alkebsi et al. found no statistically signiﬁcant difference in the rate of tooth movement between the MOP group and the control group (study duration: 3 months) [17]. This was explained by the minimal surgical insult of MOP that may not be enough to trigger the inflammatory response to activate the regional acceleratory phenomenon or even cytokine expression. The different surgical techniques employed, the unique mechanics of tooth movement explored the method of measurement, and measuring reference points are some potential explanations for the variations in results shown between research.

Comparing the mean canine retraction rate in the vibration, it was found to be almost identical (0.8mm/month), supporting the findings of Azeem et al. [18]. They investigated the effect of applying vibratory stimuli using an electric toothbrush on the rate of tooth movement. Patients were instructed to apply mechanical vibration of 125 HZ for 20 minutes daily for 90 days, and the mean rate of canine retraction was 0.81mm per month. On the other hand, a study by Pavlin et al. found that applying vibrational stimuli for 20 minutes per day at a frequency of 30 Hz improved orthodontic tooth movement [19]. The rate of tooth movement for the AccelDent group was 1.16mm/month compared to 0.79 mm/month in the control group.

The rate of space closure following maxillary premolar extraction was also studied by Leethanakul et al., and tooth movement was facilitated by vibrations from an electric toothbrush [5]. On the experimental side, he observed that tooth movement was greater than the control side (mean, 2.85±0.17 mm vs. 1.77±0.11 mm, respectively). Like Leethanakal et al. [5], An electric toothbrush was used to apply vibrations to enhance orthodontic tooth movement. The main difference was using an elastic power chain by Leethanakul et al. that was stretched between the maxillary canine and the first molar [5]. On the other hand, the Niti coil spring, which exerts reproducible and constant forces of approximately 100g, was used in the current study. This may be why the translatory movement was achieved without tipping.

As shown in table 2 mean rate of retraction of canines of Group A was more than Group B. The comparison showed a statistically significant difference between Group A and Group B. This was because MOP increases inflammatory markers by RAP that accelerates the tooth movement, whereas the vibratory stimuli do not increase the inflammatory markers. This is perhaps because the electronic toothbrush was never intended or designed to accelerate tooth movement and has insignificant potential to stimulate molecular mechanisms controlling acceleratory tooth movement.

As shown in Tables 3-4, it was observed that in Group A, out of forty canines, thirty-six (i.e., 90%) canines showed grade 0 root resorption, and four canines (i.e., 10.0%) showed grade 1 root resorption. In Group B, it was observed that out of forty canines, thirty-seven (i.e., 92.5%) canines showed grade 0 root resorption, and three canines (i.e.,7.5 %) showed grade 1 root resorption in Group B. The comparison of groups shows no statistically significant difference between Group A and Group B (p=0.694). This suggests that neither MOP nor mechanical vibrations can cause observable root resorption. However, OPG was used to evaluate root resorption, which is a two-dimensional image that could have limited the accuracy of evaluating root resorption. Similarly, a study conducted by Parihar et al. showed Grade 1 apical root resorption was noted in 37.5% of cases in the conventional group and 31.25% of cases with Grade 1, 6.25% of cases with grade 2 in the CFO+MOPs group, which was statistically not significant [15].

As shown in table 2, the mean rate of retraction of canines treated in Group A and Group B were 1.15mm per 4 weeks and 0.84mm per 4 weeks, respectively. There was a statistically significant difference between Group A and Group B. According to the study by Davis et al., the mean rate of retraction of canines with the help of TAD and conventional anchorage was 0.95mm and 0.84mm per month, respectively [20]. The retraction rate of the present study was more than that of the study conducted by Davis et al. Therefore, it is evident from the study that there was an increased rate of retraction of canines with the use of MOP compared to mechanical vibrations, thereby accelerating the orthodontic tooth movement [20].

Limitations

Patients were instructed to apply mechanical vibrations for 20 mins /day for 16 weeks so patient cooperation might have affected the study's outcome. The study only investigated the rate of retraction of canines over a 16-week period, which did not represent the entire orthodontic treatment. The current study did not evaluate the effect of different numbers, sites, and repetition of MOP on the rate of tooth movement. Measurement of canine retraction with the help of Vernier caliper is less accurate than three-dimensional superimposition. Root resorption was assessed using OPG, a two-dimensional image of a three-dimensional structure. A larger sample size was required to evaluate further the long-term effect of MOP and vibration on the tooth movement and root resorption rate.

## Conclusions

The mean rate of retraction of canine treated by MOP in young adults with bimaxillary protrusion was 1.15 mm per 4 weeks, whereas, by mechanical vibration, it was 0.84 mm per 4 weeks. The overall rate of retraction of canines treated by MOP was more than that of mechanical vibration, and a statistically significant difference was found between them. The percentage of root resorption of canines treated by MOP is more than that of canines treated by mechanical vibration. However, there was no statistically significant difference between them. MOP should be preferred in systematically healthy patients compared to non-invasive methods like mechanical vibration for accelerating orthodontic tooth movement, thereby reducing the overall treatment duration. MOP should be repeated at approximately 12-16 weeks to maintain increased inflammatory mediators in the periodontal ligament for accelerating tooth movement.

## References

[1] Alikhani M, Raptis M, Zoldan B (2013). Effect of micro-osteoperforations on the rate of tooth movement. Am J Orthod Dentofacial Orthop.

[2] Babanouri N, Ajami S, Salehi P (2020). Effect of mini-screw-facilitated micro-osteoperforation on the rate of orthodontic tooth movement: a single-center, split-mouth, randomized, controlled trial. Prog Orthod.

[3] Shingade M, Maurya R, Mishra H (2017). Accelerated orthodontics: a paradigm shift. Indian J Orthod Dentofac Res.

[4] Kannan S, Fassul S, Singh AK, Arora N, Malhotra A, Saini N (2019). Effectiveness and importance of powered tooth brushes in tooth movement. J Family Med Prim Care.

[5] Leethanakul C, Suamphan S, Jitpukdeebodintra S, Thongudomporn U, Charoemratrote C (2016). Vibratory stimulation increases interleukin-1 beta secretion during orthodontic tooth movement. Angle Orthod.

[6] Bajath B (2019). Impact of micro osteoperforations on the rate of tooth movement. J Oral Pathol.

[7] Shetty SK, Vincent S, Mahesh kumar Y (2021). Effects of micro-osteoperforations on rate of orthodontic tooth movement. Sch J Dent Sci.

[8] McNab S, Battistutta D, Taverne A (2000). External apical root resorption following orthodontic treatment. Angle Orthod.

[9] Skidmore KJ, Brook KJ, Thomson WM, Harding WJ (2006). Factors influencing treatment time in orthodontic patients. Am J Orthod Dentofacial Orthop.

[10] Shaha A (2017). Use of vibration in orthodontics. Int J of Advance Res and Development.

[11] Teixeira CC, Khoo E, Tran J (2010). Cytokine expression and accelerated tooth movement. J Dent Res.

[12] Aboalnaga AA, Salah Fayed MM, El-Ashmawi NA, Soliman SA (2019). Effect of micro-osteoperforation on the rate of canine retraction: a split-mouth randomized controlled trial. Prog Orthod.

[13] Wilcko MT, Wilcko WM, Pulver JJ, Bissada NF, Bouquot JE (2009). Accelerated osteogenic orthodontics technique: a 1-stage surgically facilitated rapid orthodontic technique with alveolar augmentation. J Oral Maxillofac Surg.

[14] Cheung T, Park J, Lee D (2016). Ability of mini-implant-facilitated micro-osteoperforations to accelerate tooth movement in rats. Am J Orthod Dentofacial Orthop.

[15] Parihar A, Verma S, Chaturvedi T (2021). Comparison of rate of canine retraction and secondary outcomes associate with conventional fixed orthodontic treatment and minimally invasive techniques: a randomized control trial. JIOS.

[16] Sivarajan S, Doss JG, Papageorgiou SN, Cobourne MT, Wey MC (2019). Mini-implant supported canine retraction with micro-osteoperforation: a split-mouth randomized clinical trial. Angle Orthod.

[17] Alkebsi A, Al-Maaitah E, Al-Shorman H, Abu Alhaija E (2018). Three-dimensional assessment of the effect of micro-osteoperforations on the rate of tooth movement during canine retraction in adults with Class II malocclusion: a randomized controlled clinical trial. Am J Orthod Dentofacial Orthop.

[18] Azeem M, Afzal A, Jawa SA Effectiveness of electric toothbrush as vibration method on orthodontic tooth movement: a split-mouth study. Dental Press J Orthod.

[19] Pavlin D, Anthony R, Raj V, Gakunga PT (2015). Cyclic loading (vibration) accelerates tooth movement in orthodontic patients: a double-blind, randomized controlled trial. Semin Orthod.

[20] Davis D, Krishnaraj R, Duraisamy S, Ravi K, Dilip S, Charles A, Sushil NC (2018). Comparison of rate of canine retraction and Anchorage potential between mini-implant and conventional molar anchorage: an in vivo study. Contemp Clin Dent.

